# Therapeutic Ultrasound Versus Levofloxacin in Lipopolysaccharide-Induced Orchitis: Comparative Effects on Testicular Inflammation and Tissue Recovery in Rats

**DOI:** 10.22034/ijp.2026.2087543.3658

**Published:** 2026-08-01

**Authors:** Nourhan Elfar, Ahmed Mamdouh Abdelkader, Doaa Sayed Mostafa, Nihal Mostafa, Sarah Sami Abdelaziz, Nadia Saad Sayed Ahmed El Gressy, Ahmed M. Atwa, Abdullah F. Radwan

**Affiliations:** 1 Biochemistry Department, Physiotherapy Program, School of Health and Social Work, University of Hertfordshire hosted by Global Academic Foundation (UH-GAF), New Administrative Capital, Cairo 11578, Egypt; 2 Integumentary Department, Physiotherapy Program, School of Health and Social Work, University of Hertfordshire hosted by Global Academic Foundation (UH-GAF), New Administrative Capital, Cairo 11578, Egypt; 3 Basic Science Department, Physiotherapy Program, School of Health and Social Work, University of Hertfordshire hosted by Global Academic Foundation (UH-GAF), New Administrative Capital, Cairo 11578, Egypt; 4 Department of Biochemistry, Faculty of Science, Ain Shams University, Cairo, Egypt; 5 Cardiorespiratory Department, Physiotherapy Program, School of Health and Social Work, University of Hertfordshire hosted by Global Academic Foundation (UH-GAF), New Administrative Capital, Cairo 11578, Egypt; 6 Teaching Fellow of Physical Therapy, El-Sahel Teaching Hospital, General Organization for Teaching Hospitals and Institutes (GOTHI), Egypt; 7 Department of Pharmacology and Toxicology, Faculty of Pharmacy, Egyptian Russian University, Cairo, Egypt; 8 College of Pharmacy, Al-Ayen Iraqi University (AUIQ), An Nasiriyah, Iraq; 9 Department of Biochemistry, Faculty of Pharmacy, Egyptian Russian University, Cairo, Egypt; 10 Department of Laboratory Science, College of Pharmacy, University of Kut, Wasit, Iraq

**Keywords:** Orchitis, therapeutic ultrasound, levofloxacin, lipopolysaccharide, testicular inflammation, rat model

## Abstract

**Background & Objective:**

Orchitis is a testicular inflammatory disorder that may impair spermatogenesis and compromise male fertility. Although fluoroquinolones are widely used in infectious orchitis and possess ancillary anti-inflammatory properties, concerns remain regarding their potential reproductive toxicity. Therapeutic ultrasound has emerged as a noninvasive modality with anti-inflammatory and tissue-modulating effects, but its role in testicular inflammation remains poorly characterized.

**Methods:**

This study evaluated the anti-inflammatory and tissue-protective effects of levofloxacin and therapeutic ultrasound in a rat model of lipopolysaccharide (LPS)-induced orchitis. Twenty-four adult male albino rats were allocated into four groups: control, untreated orchitis, levofloxacin-treated orchitis, and ultrasound-treated orchitis. Serum CRP, IL-1β, IL-6, TNF-α, IFN-γ, and IL-10 levels were measured. Testicular weight and histopathological alterations were also evaluated.

**Results:**

LPS-induced orchitis significantly increased testicular weight and proinflammatory markers while reducing IL-10 levels (P < 0.05), accompanied by marked histopathological damage. Both treatments significantly reduced IL-1β levels (P < 0.0001). Therapeutic ultrasound produced greater reductions in CRP (P < 0.01) and IFN-γ (P < 0.05) and significantly restored IL-10 levels compared with levofloxacin (P = 0.035). Levofloxacin significantly reduced testicular weight (P < 0.01), whereas ultrasound was associated with an improved histopathological appearance.

**Conclusion:**

Both interventions attenuated inflammatory alterations in the LPS-induced sterile orchitis model. Therapeutic ultrasound demonstrated more favorable cytokine modulation and histopathological findings than levofloxacin. However, the absence of a sham-ultrasound group and the lack of reproductive functional endpoints warrant cautious interpretation and further validation before clinical translation.

## Introduction

Orchitis is an inflammatory condition of the testes that can result from bacterial or viral infections, trauma, or autoimmune responses(1). Clinically, it presents with swelling, pain, and fever and, in chronic or severe cases, may lead to impaired spermatogenesis, testicular atrophy, or infertility(1,2). Bacterial orchitis is frequently associated with pathogens such as Escherichia coli, Brucella species, and sexually transmitted organisms such as Chlamydia trachomatis and Mycoplasma(3). The disease triggers a cascade of inflammatory responses, including cytokine production, oxidative stress, and disruption of the blood–testis barrier, all of which compromise testicular function and male fertility(2).

In clinical infectious orchitis, antibiotics constitute the standard therapeutic approach. However, because the present study employed an LPS-induced sterile inflammatory model rather than an active bacterial infection model, levofloxacin was included primarily as a pharmacological comparator with reported immunomodulatory properties(4). Among the antibiotics commonly used, levofloxacin, a widely used fluoroquinolone antibiotic, has gained prominence because of its broad-spectrum activity against gram-positive and gram-negative bacteria, as well as intracellular pathogens such as Mycoplasma and Chlamydia(5,6). Levofloxacin is particularly effective in treating chronic infections of the urogenital tract(7), including sexually transmitted diseases(8), brucellosis(9), and tuberculosis(10). These conditions often require prolonged courses of antibiotics, ranging from 10 to 50 days, to achieve therapeutic efficacy(11). However, accumulating evidence has highlighted potential off-target toxicities of levofloxacin, particularly involving the liver, articular cartilage, and reproductive tissues(12–15). Although its hepatotoxic and chondrotoxic effects have been relatively well characterized, its influence on testicular function, especially under inflammatory or stress-related conditions such as orchitis, remains insufficiently understood and may be detrimental(6).

In addition to pharmacological therapy, therapeutic US has emerged as a promising noninvasive modality with potential applications in male reproductive health(16). It is recognized for its localized, nonsystemic action and has been explored for tissue modulation and reversible contraceptive applications(17). Therapeutic US offers several advantages, including nonsurgical administration, ease of application, and minimal reported complications(18). Nevertheless, its long-term safety and effectiveness in testicular recovery, particularly under inflammatory conditions, remain insufficiently characterized and warrant further investigation(19–22).

The current study was designed to evaluate and compare the effects of levofloxacin and therapeutic ultrasound in an experimental rat model of LPS-induced orchitis. In addition to assessing inflammatory and histopathological alterations, the study investigated whether therapeutic ultrasound, applied under standardized physiotherapeutic parameters, could attenuate cytokine imbalance and preserve testicular tissue architecture.

The biological rationale for applying therapeutic ultrasound in orchitis is based on the inflammatory nature of testicular injury. The testis is an immune-privileged organ, but this privilege can be disrupted when inflammatory signaling becomes excessive. LPS activates innate immune pathways, particularly TLR4/NF-κB signaling, leading to increased production of inflammatory mediators that can impair Sertoli cell function, disrupt the blood–testis barrier, reduce steroidogenic activity, and compromise spermatogenesis. Experimental studies have shown that LPS-induced systemic inflammation can impair testosterone production, spermatogenesis, and blood–testis barrier integrity, with TNF-α playing a central pathogenic role. Therefore, strategies that reduce excessive inflammatory activation without adding a systemic pharmacological burden may be relevant in orchitis-associated testicular dysfunction(23,24).

Low-intensity therapeutic ultrasound has been increasingly investigated as a noninvasive biophysical intervention with anti-inflammatory and tissue-repair potential. Peer-reviewed experimental and review evidence indicates that low-intensity ultrasound can modulate inflammatory responses through pathways involving NF-κB, MAPK, PI3K/Akt, macrophage activity, and inflammatory cytokine regulation. Although direct evidence in orchitis remains limited, these mechanisms provide a reasonable basis for evaluating therapeutic ultrasound in an LPS-induced testicular inflammation model. Accordingly, the present study tested whether ultrasound could attenuate cytokine imbalance and histological injury in comparison with levofloxacin(25,26).

Given the increasing interest in nonpharmacological anti-inflammatory interventions, therapeutic ultrasound may provide a complementary strategy for reducing inflammation-associated reproductive tissue injury while minimizing prolonged systemic drug exposure.

## Materials and Methods

### Experimental Animals

Twenty-four young adult male albino rats, each weighing between 150 and 200 g, were used in this study. An a priori sample-size estimation was performed using G*Power software version 3.1.9.7(27). For one-way ANOVA with four independent groups, assuming a large expected effect size (Cohen f = 0.75), α error probability = 0.05, and statistical power = 0.80, the minimum required total sample size was 24 animals, corresponding to 6 animals per group. The selected effect size was based on the marked cytokine and histopathological alterations reported in previous LPS-induced testicular inflammation/orchitis animal studies, which commonly used approximately 5–8 animals per group. The final sample size was also selected in accordance with the ARRIVE recommendations and the ethical 3Rs principle to minimize animal use while maintaining adequate statistical power.

The animals were obtained from the Animal Core Facility of the Faculty of Agriculture, Minya University, Egypt. Prior to the initiation of the experimental protocol, all rats were acclimatized for 1 week under standard laboratory conditions in the Physiology Laboratory of the Faculty of Veterinary Medicine, Minya University. The rats were housed three per cage in polypropylene cages under a 12-hour light/dark cycle at a stable room temperature of 25 ± 2 °C, with free access to standard rodent chow and water.

After acclimatization, the animals were randomly allocated into four equal groups (n = 6 per group) as follows: Group 1, Healthy Control Group: Rats in this group received no treatment and were not subjected to testicular inflammation. This group served as the physiological baseline for all measured parameters. Group 2, Orchitis-Induced Untreated Group: Rats in this group were injected intraperitoneally with lipopolysaccharide (LPS) to induce testicular inflammation (orchitis), but no therapeutic intervention was provided. This group was used to observe the natural progression of LPS-induced inflammation. Group 3, Levofloxacin-Treated Orchitis Group: Rats were subjected to LPS-induced orchitis as in Group 2 and subsequently treated with levofloxacin, which was administered subcutaneously at a dose of 200 mg/kg body weight. The injection volume was adjusted individually according to each animal’s body weight. Two doses were administered 48 hours apart. Group 4, US-Treated Orchitis Group: Following induction of orchitis with LPS, rats in this group were treated with therapeutic US.

Rats were randomly allocated to the four experimental groups using a simple randomization procedure. Owing to the nature of the interventions, blinding during treatment administration was not feasible. However, serum samples and histological sections were coded prior to analysis whenever possible to minimize potential bias. Histopathological evaluation was performed by an experienced pathologist who was blinded to group allocation. No formal allocation concealment procedure was implemented.

### Therapeutic Ultrasound Procedure

Therapeutic ultrasound was applied using the Primo Combination 860 physiotherapy device (S.A.G. International, Nasr City, Cairo, Egypt) (Supplementary Figs. S1 and S2). Treatment was performed via underwater coupling to ensure uniform acoustic transmission and minimize skin irritation. The transducer, with a 3-cm² effective radiating area, operated at a frequency of 1 MHz and delivered a continuous wave at 1.5 W/cm². Rats were gently positioned in a shallow water bath to expose the scrotal region, and the probe was maneuvered in slow, circular motions approximately 1 cm above the testicular area, ensuring no direct contact. Each session lasted 8 minutes and was conducted 2–3 times per week over a 4-week period, totaling 10 sessions. This physiotherapeutic regimen was designed to modulate local inflammation, improve microvascular perfusion, and support tissue preservation without causing thermal damage.

The ultrasound protocol was adapted from established therapeutic ultrasound principles commonly used in experimental soft-tissue and inflammatory studies. Underwater coupling was employed to enhance acoustic transmission and minimize direct mechanical pressure on the scrotal surface, while continuous circular movement of the transducer was maintained to distribute acoustic energy evenly and reduce focal energy accumulation. A frequency of 1 MHz was selected because it provides adequate penetration into deeper soft tissues while limiting excessive superficial energy deposition. In addition, the selected intensity and exposure duration were maintained within ranges previously reported in experimental therapeutic ultrasound studies investigating inflammatory modulation and tissue preservation without evidence of overt thermal injury(28–32). Although the ultrasound device was originally developed for clinical physiotherapy applications, the protocol was carefully standardized for the rat model through controlled adjustment of ultrasound frequency, intensity, probe distance, exposure duration, coupling method, and treatment frequency to ensure reproducibility and biological safety in small experimental animals(28,30,32).

### Preparation of Levofloxacin

Levofloxacin used in this study was obtained in tablet form (Levoxin 500 mg, Amoun Pharmaceutical Co SAE, El-Obour City, Cairo, Egypt). Each tablet was finely ground and dissolved in 25 mL of normal saline to prepare a 2% solution suitable for subcutaneous administration. The solution was freshly prepared before administration, and the administered volume was calculated according to the body weight of each rat.

Levofloxacin was administered subcutaneously at a dose of 200 mg/kg in two doses separated by 48 hours. The selected dose and administration schedule were adapted from previously published experimental studies and from the classical work of Gough et al, which demonstrated reproducible fluoroquinolone-induced tissue injury in rodents. Because injectable-grade levofloxacin was unavailable, a tablet formulation was suspended in sterile normal saline immediately before administration(33,34).

### Testicular Weight Measurement

At the end of the experimental period, all animals were weighed using a calibrated digital balance to determine their final body weight. Subsequently, the animals were euthanized under appropriate anesthesia, and their testes were carefully excised and immediately weighed using a high-precision analytical balance. Testicular weights were recorded individually for each animal in milligrams for each experimental group. This measurement was used to assess the effects of disease induction and therapeutic interventions on testicular mass as an indicator of reproductive organ integrity.

### Induction of Orchitis

Lipopolysaccharide was administered as a single intraperitoneal dose of 5 mg/kg body weight at the beginning of the experiment(35). Inflammatory responses were allowed to become established over the subsequent 24–48 hours before initiation of the therapeutic interventions.

### Determination of Serum CRP, IL-6, IL-1β, IL-10, TNF-α, and IFN-γ

The following kits were used: Rat CRP (C-Reactive Protein ELISA Kit, Cat. No. E-EL-R0022); Rat IL-1β (Interleukin 1 Beta ELISA Kit, Cat. No. E-EL-R0012); Rat IL-6 (Interleukin 6 ELISA Kit, Cat. No. E-EL-R0015); Rat IL-10 (Interleukin 10 ELISA Kit, Cat. No. E-EL-R0016); Rat IFN-γ (Interferon Gamma ELISA Kit, Cat. No. E-EL-R0009); and Rat TNF-α (Tumor Necrosis Factor Alpha ELISA Kit, Cat. No. E-EL-R0019). Rat ELISA kits for detecting CRP, IL-6, IL-1β, IL-10, TNF-α, and IFN-γ were purchased from Elabscience Biotechnology Co, Ltd, Building 4, Room 403, Guandong Science and Technology Industry Park, Wuhan, People’s Republic of China.

### Equipment

The equipment used included a Stat Fax 2100 microplate reader (Awareness Technology Inc, PO Drawer 1679, Palm City, FL 34991, USA); an OLYMPUS laboratory trinocular LED microscope, model CX23, with a digital camera from Optoscient; a Primo Combination 860 ultrasound therapy unit purchased from S.A.G. International for Medical Equipment, Nasr City, Cairo, Egypt; and a Mettler Toledo analytical balance (XS204, Columbus, OH, USA).

### Histopathological Preparations

The testes were removed, washed, and then fixed in 10% formalin. The fixed tissue samples were dehydrated using ascending concentrations of ethanol (70%, 80%, 90%, 100%, and 100%). The samples were embedded in paraffin blocks and sectioned at 6 µm using a rotary microtome. Sections were stained with hematoxylin-eosin. Slides were examined using a light microscope (PANTHERA E2, Motic, Hong Kong) equipped with a camera (Moticam A16). Photomicrographs from each animal were recorded using a 10× objective lens in 15 different fields.

### Statistical Methods

Data are presented as mean ± standard error of the mean (SEM). Normality was assessed using the Shapiro–Wilk test. Normally distributed data were analyzed using one-way ANOVA followed by Tukey multiple comparisons test using GraphPad Prism version 9.5 (GraphPad Software, California, USA). Statistical significance was set at P < 0.05.

## Results

### Effect of Levofloxacin and Ultrasound on Testicular Weight

**Fig. 1:**
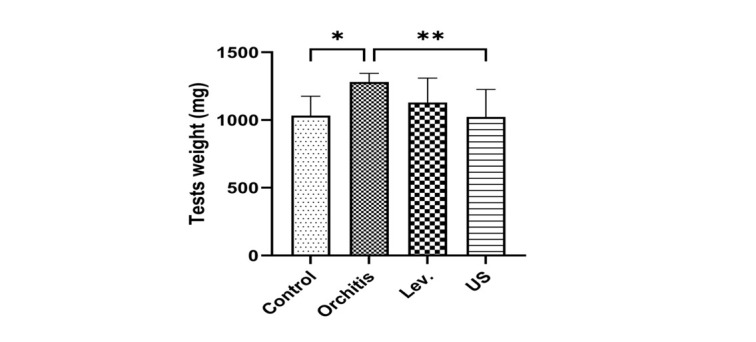
Effect of levofloxacin and therapeutic ultrasound on testicular weight in LPS-induced orchitis. Data are presented as mean ± SEM (n = 6 per group). Statistical analysis was performed using one-way ANOVA followed by Tukey's multiple comparisons test. Horizontal bars indicate the specific pairwise comparisons shown. *P < 0.05 and **P < 0.01.

As shown in Fig. 1, testicular weight was significantly increased in the orchitis-induced group compared with the healthy control group (P < 0.05). Administration of levofloxacin resulted in a significant reduction in testicular weight compared with the orchitis group (P < 0.01). The US-treated group showed no statistically significant difference in testicular weight compared with either the orchitis or control groups. Data are presented as mean ± SEM (n = 6 per group).

### Effect of Levofloxacin and Ultrasound on Serum Inflammatory Markers in a Rat Model of Orchitis

**Fig. 2:**
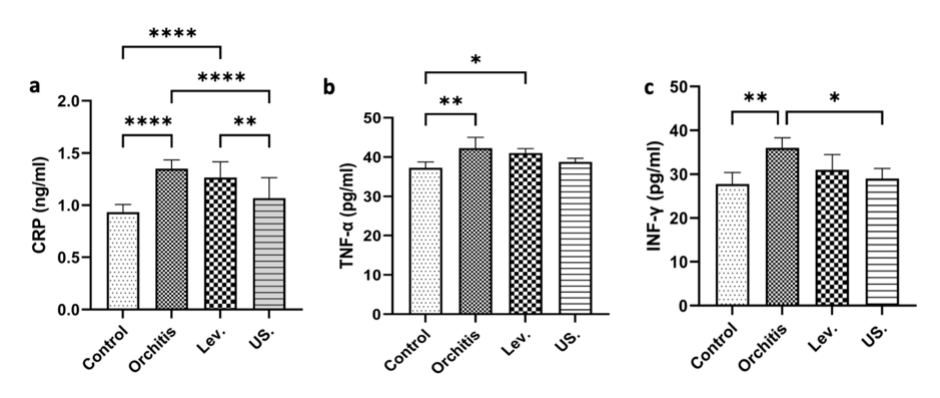
Effects of levofloxacin and therapeutic ultrasound on systemic inflammatory markers in LPS-induced orchitis. Serum levels of (a) C-reactive protein (CRP), (b) tumor necrosis factor-α (TNF-α), and (c) interferon-γ (IFN-γ) were measured across experimental groups. Data are expressed as mean ± SEM (n = 6 per group). Statistical analysis was performed using one-way ANOVA followed by Tukey's multiple comparisons test. Horizontal bars indicate the specific pairwise comparisons shown. *P < 0.05, **P < 0.01, and ****P < 0.0001.

As shown in Fig. 2a and Table 1, CRP levels were significantly increased in the orchitis-induced group compared with the healthy control group (1.351 ± 0.082 vs 0.9325 ± 0.074 ng/mL, P = 0.0194). Levofloxacin-treated rats showed numerically lower CRP concentrations than untreated orchitis animals (1.267 ± 0.148 ng/mL); however, the difference did not reach statistical significance. In contrast, therapeutic ultrasound significantly reduced CRP levels compared with the untreated orchitis group (1.068 ± 0.196 vs 1.351 ± 0.082 ng/mL, P = 0.008), as shown in Fig. 2a and Table 1, with detailed pairwise comparisons provided in Supplementary Table S1.

**Table 1:** Serum Levels of Inflammatory and Anti-Inflammatory Biomarkers in the Studied Groups

Parameter	Control (Mean ± SEM)	Orchitis (Mean±SEM)	Levofloxacin (Mean ± SEM)	Ultrasound (Mean ± SEM)	95% CI	Adjusted P-value	Pairwise comparison	Overall ANOVA P-value
CRP (ng/mL)	0.9325 ± 0.074	1.351 ± 0.082	1.267 ± 0.148	1.068 ± 0.196	-461.4 to -31.52 -298.5 to 108.0 -184.2 to 205.9 -58.61 to 361.0 55.45 to 459.2 -83.25 to 295.5	0.0194 0.5914 0.9988 0.2289 0.008 0.4417	A-B A-C A-D B-C B-D C-D	0.0194
IL-1β (pg/mL)	21.3 ± 2.406	27.65 ± 2.143	22.68 ± 2.18	22.5 ± 2.173	-9.030 to -3.662 -4.066 to 1.302 -3.884 to 1.484 2.280 to 7.648 2.462 to 7.830 -2.502 to 2.866	<0.0001 0.5155 0.6281 <0.0001 <0.0001 0.9978	A-B A-C A-D B-C B-D C-D	<0.0001
IL-6 (pg/mL)	35.89 ± 3.516	42.67 ± 2.179	40.44 ± 1.944	38.11 ± 1.9	-9.939 to -3.617 -7.716 to -1.395 -5.383 to 0.9387 -0.9387 to 5.383 1.395 to 7.716 -0.8276 to 5.494	<0.0001 0.0025 0.2463 0.2463 0.0025 0.2092	A-B A-C A-D B-C B-D C-D	<0.0001
IL-10 (pg/mL)	108 ± 8.617	94 ± 3.162	97.22 ± 3.563	105.6 ± 7.468	6.108 to 21.89 2.886 to 18.67 -5.447 to 10.34 -11.11 to 4.670 -19.45 to -3.664 -16.23 to -0.4414	0.0002 0.0043 0.8354 0.6884 0.0021 0.0353	A-B A-C A-D B-C B-D C-D	0.0002
TNF-α (pg/mL)	37.25 ± 1.5	42.25 ± 2.754	41 ± 1.155	38.75 ± 0.9574	-8.649 to -1.351 -7.399 to -0.1012 -5.149 to 2.149 -2.399 to 4.899 -0.1488 to 7.149 -1.399 to 5.899	0.0073 0.0434 0.6264 0.7429 0.0616 0.3067	A-B A-C A-D B-C B-D C-D	0.0073
IFN-γ (pg/mL)	27.75 ± 2.63	36 ± 2.309	31 ± 3.464	29 ± 2.309	-13.96 to -2.541 -8.959 to 2.459 -6.959 to 4.459 -0.7092 to 10.71 1.291 to 12.71 -3.709 to 7.709	0.005 0.37 0.9135 0.0936 0.0155 0.7302	A-B A-C A-D B-C B-D C-D	0.005

TNF-α levels were significantly higher in the orchitis group than in controls (42.25 ± 2.754 vs 37.25 ± 1.5 pg/mL, P = 0.0073). Levofloxacin-treated rats showed a numerically lower TNF-α level than the untreated orchitis group (41 ± 1.155 pg/mL); however, this reduction did not reach statistical significance (P = 0.7429 vs orchitis). Similarly, ultrasound-treated rats exhibited lower TNF-α levels (38.75 ± 2.63 pg/mL), although the difference compared with the untreated orchitis group did not reach statistical significance (P = 0.0616), as shown in Fig. 2b and Table 1, with detailed pairwise comparisons provided in Supplementary Table S5.

Regarding IFN-γ, the orchitis group demonstrated significantly elevated levels relative to the control group (36 ± 2.309 vs 27.75 ± 2.63 pg/mL, P = 0.005). Ultrasound treatment significantly reduced IFN-γ levels compared with the untreated orchitis group (29 ± 2.309 pg/mL, P = 0.0155). In contrast, levofloxacin-treated rats showed a numerically lower IFN-γ level (32 ± 2.582 pg/mL); however, the difference compared with the orchitis group did not reach statistical significance (P = 0.0936). No significant difference was observed between the levofloxacin and ultrasound groups (P = 0.7302), as shown in Fig. 2c and Table 1, with detailed pairwise comparisons provided in Supplementary Table S6.

**Fig. 3:**
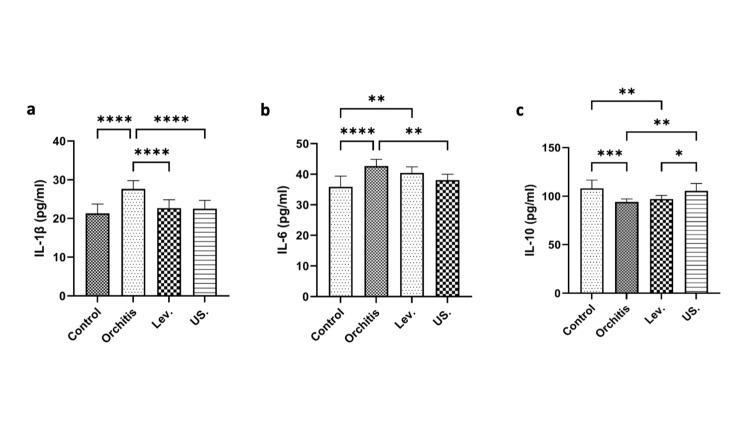
Effects of levofloxacin and therapeutic ultrasound on pro- and anti-inflammatory cytokines in LPS-induced orchitis. Serum levels of (a) interleukin-1β (IL-1β), (b) interleukin-6 (IL-6), and (c) interleukin-10 (IL-10) were evaluated. Data are presented as mean ± SEM (n = 6 per group). Statistical analysis was performed using one-way ANOVA followed by Tukey's multiple comparisons test. Horizontal bars indicate the specific pairwise comparisons shown. *P < 0.05, **P < 0.01, ***P < 0.001, and ****P < 0.0001.

As shown in Fig. 3a and Table 1, serum IL-1β concentrations were significantly elevated in the orchitis group compared with controls (27.65 ± 2.143 vs 21.3 ± 2.406 pg/mL, P < 0.0001). Both levofloxacin- and ultrasound-treated rats exhibited significantly lower IL-1β levels than the untreated orchitis group (22.68 ± 2.18 and 22.5 ± 2.173 pg/mL, respectively; P < 0.0001 for both). No significant difference was observed between the levofloxacin and ultrasound groups (P = 0.9978), as shown in Fig. 3a and Table 1, with detailed pairwise comparisons provided in Supplementary Table S2.

Similarly, IL-6 levels were significantly higher in the orchitis group than in controls (42.67 ± 2.179 vs 35.89 ± 3.516 pg/mL, P < 0.0001). Levofloxacin-treated rats showed numerically lower IL-6 levels (40.44 ± 2.121 pg/mL); however, the difference compared with the untreated orchitis group did not reach statistical significance (P = 0.2463). In contrast, ultrasound treatment significantly reduced IL-6 levels compared with the untreated orchitis group (38.11 ± 1.9 vs 42.67 ± 2.179 pg/mL, P = 0.0025). No significant difference was observed between the levofloxacin and ultrasound groups (P = 0.2092), as shown in Fig. 3b and Table 1, with detailed pairwise comparisons provided in Supplementary Table S3.

For IL-10, a notable decrease was observed in the orchitis group compared with controls (94 ± 3.162 vs 108 ± 8.617 pg/mL, P = 0.0002). Levofloxacin-treated rats showed a numerical increase in IL-10 levels (97.22 ± 3.563 pg/mL); however, the difference compared with the orchitis group was not statistically significant (P = 0.6884). In contrast, ultrasound therapy significantly increased IL-10 levels relative to the untreated orchitis group (105.6 ± 7.468 pg/mL, P = 0.0021). Moreover, IL-10 levels were significantly higher in the ultrasound group than in the levofloxacin group (P = 0.0353), as shown in Fig. 3c and Table 1. Detailed pairwise comparisons are provided in Supplementary Table S4.

### Histopathological Findings

**Fig. 4:**
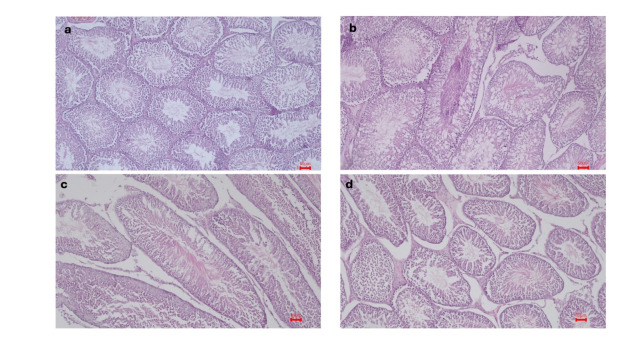
Histopathological evaluation of testicular tissue across experimental groups (H&E staining, 10× magnification; scale bar = 50 µm). (a) Control group showing normal seminiferous tubules with intact architecture and preserved germinal epithelium. (b) Orchitis group demonstrating marked tubular degeneration, vacuolization, loss of germinal epithelium, and interstitial edema. (c) Levofloxacin-treated group showing partial improvement of testicular architecture with reduced inflammatory changes. (d) Ultrasound-treated group exhibiting improved histological appearance with better preservation of seminiferous tubule organization and minimal inflammatory changes.

Histological evaluation of testicular tissue revealed distinct pathological alterations across the experimental groups. In Group 1, as shown in Fig. 4a, representing the healthy control group, seminiferous tubules were uniformly arranged and morphologically intact. The germinal epithelium was multilayered, with clearly distinguishable spermatogonia, spermatocytes, and spermatids. The lumen contained maturing spermatozoa, and the interstitial space appeared normal and free from edema or inflammatory infiltration. In contrast, Group 2, shown in Fig. 4b, which received no treatment after induction of testicular inflammation, exhibited severe degenerative changes. Seminiferous tubules appeared distorted and shrunken, with marked disorganization of the germinal epithelium, extensive vacuolization, and loss of spermatogenic cell layers. The interstitium showed prominent edema, vascular congestion, and moderate inflammatory infiltration, confirming acute orchitis.

Group 3, shown in Fig. 4c and treated with levofloxacin following orchitis induction, demonstrated moderate structural preservation. Several seminiferous tubules retained partial epithelial organization, and some showed partial preservation of spermatogenic cell layers, although maturation was incomplete. Interstitial edema was reduced, but mild inflammatory cell infiltration persisted in focal areas. Group 4, shown in Fig. 4d, which received therapeutic US, showed marked improvement in histopathological appearance. The seminiferous tubules appeared well preserved and rounded, with improved histological appearance and preserved spermatogenic cell layers. The interstitial compartment was largely unremarkable, with minimal signs of inflammation or edema. These findings suggest improved preservation of seminiferous tubule architecture in the ultrasound-treated group compared with the levofloxacin-treated group.

This table summarizes the serum concentrations (mean ± SEM) of C-reactive protein (CRP), proinflammatory cytokines (IL-1β, IL-6, TNF-α, and IFN-γ), and the anti-inflammatory cytokine IL-10 across four experimental groups: healthy control, orchitis-induced untreated, levofloxacin-treated, and ultrasound-treated rats. Overall P values represent one-way ANOVA results for comparisons among all groups. Pairwise comparisons between individual groups were performed using Tukey multiple comparisons test following one-way ANOVA.

## Discussion

Testicular inflammation, or orchitis, is a significant contributor to male infertility, often arising from bacterial infections or systemic inflammatory responses(36). Experimental animal models are crucial for studying the pathological mechanisms involved and for testing therapeutic strategies(37). Among these, LPS-induced orchitis is a commonly used and well-validated model, as it replicates key features of infection-related testicular inflammation observed in clinical settings(38). LPS is a potent endotoxin derived from the outer membrane of gram-negative bacteria and initiates inflammation by activating Toll-like receptor 4 (TLR4), leading to the release of cytokines such as TNF-α and IL-6(39). This cascade disrupts the blood–testis barrier, impairs Sertoli cell function, and causes degeneration of the seminiferous epithelium(39,40). In addition, histopathological evaluation following LPS administration reveals acute testicular injury characterized by germ cell loss, epithelial disorganization, and interstitial edema(41). These morphological alterations are consistent with cytokine-mediated damage and highlight the mechanism by which inflammation compromises spermatogenesis(23). Furthermore, a significant increase in testicular weight was observed in the orchitis group compared with healthy controls, attributable to inflammation-induced edema(42). This measurable physical change supports the effectiveness of LPS in inducing a robust and reproducible inflammatory response, confirming the validity of this model for assessing therapeutic interventions targeting testicular inflammation(23,40–42).

Importantly, the present model represents endotoxin-induced sterile inflammation rather than active bacterial orchitis, and rodent inflammatory models should be interpreted accordingly(4). Therefore, the study was not intended to evaluate antibacterial efficacy or to compare therapeutic ultrasound with standard antimicrobial treatment. Instead, levofloxacin was included as a pharmacological comparator because fluoroquinolones have been reported to exert immunomodulatory effects beyond their antimicrobial activity through modulation of inflammatory signaling pathways. Consequently, the observed differences between interventions should be interpreted within the context of inflammatory regulation in an experimental setting rather than as evidence of superiority over conventional clinical management of infectious orchitis(38).

This study aimed to evaluate and compare the therapeutic effects of levofloxacin, a widely prescribed fluoroquinolone antibiotic, and therapeutic US, a nonpharmacological physiotherapeutic modality, on testicular inflammation and function in a rat model of LPS-induced orchitis. By assessing the systemic immune response and the efficacy of each intervention, serum levels of key proinflammatory cytokines (IL-1β, IL-6, TNF-α, and IFN-γ) were measured, alongside CRP and the anti-inflammatory cytokine IL-10. In addition, testicular weight changes and histopathological alterations were evaluated to provide a comprehensive assessment of testicular tissue integrity and inflammatory status.

Starting with the inflammatory biomarkers, CRP levels were significantly elevated in the orchitis group compared with the control group, indicating a marked systemic inflammatory response. This is consistent with previous findings showing that LPS exposure leads to significant increases in CRP and proinflammatory cytokines through activation of the TLR4/NF-κB pathway(43). Levofloxacin-treated animals showed numerically lower CRP levels; however, the reduction did not reach statistical significance, and CRP values remained above those observed in healthy controls. This observation is consistent with previous reports suggesting that fluoroquinolones may provide only limited resolution of inflammation, particularly in immune-privileged organs such as the testis(44). In contrast, therapeutic US achieved a significant decrease in CRP levels, bringing values closer to baseline. This finding is supported by studies demonstrating that low-intensity pulsed US can reduce systemic inflammatory markers, including CRP, through modulation of local cytokine expression and enhancement of microvascular circulation(45,46). Collectively, these findings suggest that therapeutic ultrasound may provide broader immunomodulatory effects in the context of LPS-induced orchitis.

Immunologically, the orchitis group displayed a classical proinflammatory cytokine profile, characterized by elevated levels of IL-1β, IL-6, and IFN-γ, all key mediators of innate immune activation and testicular damage. These cytokines are known to contribute to blood–testis barrier disruption, germ cell apoptosis, and immune cell infiltration during LPS-induced inflammation(38,47). Levofloxacin significantly decreased IL-1β, suggesting partial suppression of inflammasome activation and neutrophilic infiltration. This aligns with previous findings reporting that fluoroquinolones can attenuate IL-1β expression. These findings indicate that levofloxacin’s immunomodulatory effect may be limited to specific inflammatory pathways and may be insufficient to fully attenuate the broader cytokine cascade associated with endotoxin-induced testicular injury(4,48,49).

The comparatively limited effect of levofloxacin on broader cytokine restoration may reflect the difference between antimicrobial activity and direct immunomodulation. Although fluoroquinolones possess partial anti-inflammatory properties, their primary mechanism remains antibacterial rather than tissue-regenerative. In inflammatory conditions driven predominantly by endotoxin-mediated cytokine activation rather than active bacterial proliferation, suppression of inflammatory signaling may require broader immunoregulatory modulation beyond antimicrobial intervention alone(4,44,49).

In contrast, therapeutic US significantly reduced all three key proinflammatory mediators, IL-1β, IL-6, and IFN-γ, indicating a broader anti-inflammatory effect. The broader anti-inflammatory profile observed following ultrasound treatment may be related to previously reported immunomodulatory effects of low-intensity ultrasound on inflammatory signaling pathways and cytokine regulation. However, because mechanistic endpoints such as TLR4/NF-κB activation, macrophage polarization markers, and tight-junction proteins were not directly assessed in the present study, these mechanisms should be interpreted as biologically plausible rather than experimentally confirmed(25,26,31,32,50).

In addition to reducing CRP, IL-1β, IL-6, and IFN-γ, ultrasound significantly restored IL-10 levels, indicating partial restoration of inflammatory balance. Previous studies have similarly reported that low-intensity ultrasound can attenuate LPS-induced inflammatory responses through modulation of NF-κB-related signaling pathways(26,51).

Notably, US also significantly increased IL-10, a potent anti-inflammatory cytokine that plays a central role in tissue protection, immune regulation, and suppression of Th1-driven immune responses. IL-10 is also known to inhibit IL-6 and TNF-α synthesis, which may explain the improved CRP levels and preservation of testicular architecture in the US group. These findings are supported by evidence showing that therapeutic US can enhance IL-10 expression in inflamed tissues via modulation of the JAK/STAT and PI3K/Akt pathways, thereby promoting immune resolution(52–54). Taken together, these findings demonstrate that levofloxacin exerts a partial anti-inflammatory effect, predominantly through IL-1β inhibition, but may be inadequate to fully counteract the cytokine cascade triggered by LPS. In contrast, therapeutic US offers a more comprehensive immunomodulatory profile, simultaneously downregulating damaging inflammatory mediators and promoting regulatory cytokine activity. These findings underscore the potential of US as a nonpharmacological immunomodulatory approach for attenuating inflammation-associated testicular injury(55).

Oxidative stress is another major contributor to inflammation-associated testicular dysfunction and may partially explain the observed histopathological alterations. Previous studies have demonstrated that LPS-induced orchitis is associated with increased reactive oxygen species generation, lipid peroxidation, and disruption of antioxidant defense systems within testicular tissue(2,29). Likewise, fluoroquinolone-associated reproductive toxicity has been linked to oxidative damage and mitochondrial dysfunction(14). Although oxidative stress markers were not evaluated in the current study, the observed histological preservation following ultrasound therapy may partly relate to previously reported anti-inflammatory and tissue-reparative effects of low-intensity ultrasound on oxidative tissue injury(49,56). Future studies incorporating oxidative stress biomarkers would therefore provide additional mechanistic insight into ultrasound-mediated testicular protection.

Regarding testicular weight, levofloxacin significantly reduced the orchitis-induced increase in testicular weight, suggesting a reduction in inflammation-associated edema. In contrast, the ultrasound-treated group showed no significant difference compared with either the orchitis or control groups, indicating preservation of testicular mass while improving tissue architecture. This finding suggests that ultrasound may promote structural recovery without markedly affecting gross organ weight, whereas levofloxacin appears to reduce inflammation-associated swelling. This observation is supported by previous studies reporting that changes in testicular weight do not always parallel histopathological alterations, indicating that gross organ weight may not immediately reflect cellular-level damage or recovery(44,57,58).

The histopathological findings from this study provide compelling evidence for the differential impact of levofloxacin and therapeutic US on inflammation-induced testicular damage. In line with prior studies, induction of orchitis using LPS resulted in acute testicular injury characterized by germ cell loss, epithelial disorganization, and interstitial edema(35). These morphological alterations are consistent with the inflammatory cascade known to disrupt the blood–testis barrier and impair spermatogenesis through cytokine-mediated damage(58). Levofloxacin, despite its broad-spectrum antibacterial efficacy, demonstrated only moderate protective effects in testicular tissue. Although partial recovery of the seminiferous epithelium was observed, residual vacuolation and inflammatory infiltration persisted, consistent with reports of fluoroquinolone-associated testicular toxicity under inflammatory stress(44). In contrast, therapeutic US markedly improved the histological appearance of testicular tissue, with preservation of epithelial integrity and better preservation of seminiferous tubule organization compared with the untreated orchitis group. These findings are supported by previous experimental models in which scrotal US was shown to reduce inflammation, enhance local circulation, and improve the histological appearance of testicular tissue(31,32,56). The mechanical and thermogenic effects of US likely contributed to both the attenuation of inflammatory responses and the improvement of tissue organization(50,59,60). Therefore, the data suggest that while both interventions offer protective benefits, US therapy was associated with more favorable histopathological findings, supporting its potential as a noninvasive adjunctive approach for inflammation-associated testicular injury.

Overall, this study provides a novel comparative analysis of levofloxacin and therapeutic US in an established rat model of lipopolysaccharide-induced orchitis, offering valuable insights into the biological effects of both interventions on testicular inflammation. One of the major strengths of the study lies in the dual assessment approach, combining quantitative biochemical markers (eg, CRP and inflammatory cytokines) with histological evaluation to provide a more comprehensive understanding of the testicular tissue response. Moreover, the inclusion of both proinflammatory and anti-inflammatory cytokines (IL-1β, IL-6, TNF-α, IFN-γ, and IL-10) offers a nuanced characterization of the immunological landscape following treatment. Another strength is the implementation of a physiotherapeutically calibrated ultrasound protocol using standardized frequency, intensity, and session duration parameters, which enhances the translational relevance of the findings for future nonpharmacological interventions.

Despite these strengths, several limitations should be acknowledged. First, the sample size per group was relatively small (n = 6), which may limit statistical power and generalizability. Second, the study assessed short-term outcomes over a 4-week period without evaluating long-term effects on spermatogenesis, fertility, or hormonal profiles, which are crucial for assessing true reproductive recovery. Third, while US treatment showed favorable cytokine modulation, its precise cellular and molecular mechanisms remain speculative and warrant further mechanistic investigation.

An important methodological limitation of the present study is the absence of a sham-ultrasound control group. Consequently, nonspecific effects related to animal handling, repeated water-bath exposure, procedural stress, or placebo-like environmental influences cannot be completely excluded. Therefore, although cytokine modulation and histological improvement were observed, the specific contribution attributable solely to ultrasound treatment should be interpreted with caution. In addition, although histological recovery and cytokine modulation were demonstrated, the study did not evaluate sperm parameters, testosterone levels, oxidative stress markers, or fertility outcomes. Consequently, the observed tissue-protective effects cannot yet be directly translated into confirmed reproductive functional recovery. Future sham-controlled mechanistic studies incorporating reproductive, hormonal, and molecular endpoints are warranted to validate the therapeutic relevance of ultrasound in inflammatory testicular injury.

From a clinical perspective, these findings support further investigation of therapeutic ultrasound as a potential adjunctive strategy for inflammation-associated testicular injury. Such an approach may be particularly relevant in conditions characterized by persistent inflammation or in situations where prolonged pharmacological treatment may be undesirable. Theoretically, the present study contributes to the growing body of literature exploring biophysical therapies for inflammatory and reproductive disorders, thereby expanding the current understanding of testicular immunophysiology.

Another limitation of the present study is the use of a tablet formulation of levofloxacin because injectable-grade preparations were unavailable. Consequently, the potential contribution of tablet excipients could not be completely excluded. Furthermore, no dedicated vehicle-control group was included. Future investigations employing injectable formulations, appropriate vehicle controls, and additional toxicological assessments are warranted to further strengthen the interpretation of these findings.

Collectively, the present findings support the concept that biophysical immunomodulation may represent a complementary strategy for inflammatory reproductive disorders, particularly in settings where prolonged antibiotic exposure, incomplete inflammatory resolution, or reproductive toxicity remain clinical concerns.

## Conclusion

This study demonstrates that, within the context of an LPS-induced sterile inflammatory model, therapeutic ultrasound exerted broader immunomodulatory effects and was associated with more favorable histopathological findings than those observed in the levofloxacin-treated group. While levofloxacin partially attenuated inflammatory alterations, ultrasound therapy more effectively modulated inflammatory cytokine profiles and was associated with an improved histological appearance of testicular tissue. These findings should not be interpreted as evidence against antibiotic therapy in infectious orchitis but rather support the potential role of therapeutic ultrasound as an adjunctive anti-inflammatory strategy for inflammation-associated testicular injury.

Importantly, the present findings are limited to inflammatory biomarkers, testicular weight, and histopathological observations and should not be interpreted as evidence of functional reproductive recovery or restoration of fertility.

Nevertheless, the absence of a sham-ultrasound control group represents an important limitation of the present study. Therefore, future sham-controlled investigations incorporating sperm analysis, hormonal assessment, fertility outcomes, oxidative stress markers, and molecular endpoints are required before clinical translation can be established.

## Data Availability

The manuscript contains all of the data and materials used.
